# Progress in Surgery

**Published:** 1898-07-30

**Authors:** 


### Progress in Surgery.

### GENITO-URINARY SURGERY.

(*Continued from page* 290.)

Calculous anuria is only amenable to operative treatment, which gives a patient a chance of life. The symptoms are at first insidious without uræmia. Uræmia sets in about the seventh or eighth day of suppression, marked vomiting being a prominent feature, with contracted pupils and muscular tremors. Operation should be performed as soon as the diagnosis is made from the history, the examination of the abdomen, vagina, rectum, &c. The rule should be to operate on the side last affected when the indication on this point is clear.

As regards the technique of operations on the kidney and ureter the bladder may first be sounded and the lower end of the ureter be palpated per vaginum or per rectum. The kidney should be brought on to the loin and the pedicle temporarily clamped with the fingers. Needling is unreliable, so an incision in the convex border of the kidney should be made with a scalpel, and the finger inserted. The ureter should be probed to see that it is quite free. After removal of the calculus the renal wound should be closed with sutures, and a drainage tube be passed to, but not into the kidney.

An interesting case of early tuberculosis of a kidney with stricture of the ureter is given by Dr. Vineberg. The kidney was successfully extirpated.[7] One hundred and thirty-five cases of tuberculous kidney have been collected by Dr. Bolton Bangs (New York). Of these 20 per cent. died within a month of operation, whilst 56 per cent. were promising survivors of from one month to eight years.

Dr. A. F. Jonas (Omaha)[8] reports a successful extirpation of the suprarenal capsule for tuberculous disease, and it is interesting to note that the bronzing of the skin subsequently disappeared. If, therefore, early diagnosis can be made, and if it can be determined which adrenal is diseased in the first instance then there is possibility of surgical cure.

**Surgery of the Renal Pelvis and Ureters.**—Catheterisation of the uterers in the male appears to have been rendered very simple and comparatively easy by Dr. Howard Kelly, of Baltimore.[9] He uses straight tubes similar to those employed for the female, but longer; and by placing the patient in the knee-elbow position, and raising the penis backwards between the buttocks, a straight tube with obturator can be inserted. The special points are that the rectum is first distended with air; and then, when the obturator of the speculum is removed, the bladder also becomes inflated. This method would appear to open up a new and important departure in cystoscopy and ureteral catheterisation.

The operations on the ureters have been thoroughly detailed by Henry Morris in his Hunterian Lectures.[10] Of those on the pelvis of the kidney, an ingenious method is figured, whereby a valvular orifice to the ureter can be remedied by making a transverse slit in the pelvis and sewing this up vertically. Owing to the liability of the ureter to injury, surgeons should be acquainted with the methods of ureteral anastomosis, both lateral and end to end. The urethra is capable of much increase in lumen by stretching. After the anastomosis, the ureter should be enveloped in a fold of peritoneum. Those interested in this subject should refer to the reports of the lectures themselves. Mr. Betham Robinson has described two cases in which he removed calculi from the ureter with success.[11]

**The Prostate.**—Dr. Willy Meyer speaks highly of the value of Nitze's cystoscope in the diagnosis of hypertrophied prostate.[12] Dr. Bolton Bangs holds that the first steps in enlargement of the prostate take place in youth.[13] He also emphasises the fact that "the first introduction of a catheter into the bladder of a patient should be regarded as a surgical operation;" and the most scrupulous care should be taken to avoid infection of the bladder, both on the part of the surgeon, and by careful tuition on the part of the patient.

In the case of tubercular affection of the vas deferens, Dr. Robert Weir speaks well of avulsion of the cord at the same time as castration.[14] Union of a divided vas may be successfully accomplished, but always with an obstructed lumen. Dr. Willy Meyer speaks favourably of his experience of Bottini's operation for hypertrophied prostate, and says it "decidedly deserves to be given a careful and unbiassed test." One great advantage is that it removes the obstruction without mutilating other parts.[15]

**Bladder, &c.**—A successful operation for a hernia of the bladder is detailed by Mr. Henry Thompson, who applied a serre-nœud and cut off the redundant sac.[16]

The detection of stone in the presence of prostatic hypertrophy may be difficult. Dr. Horwitz (Philadelphia) gives three cases in which he performed double castration to reduce the prostate, and to make an examination of the bladder possible.[17]

Dr. P. J. Freyer speaks highly of the value of an evacuator, and also of the cystoscope, in the diagnosis of small vesical calculi.[18] Total extirpation of the bladder for cancer has been successfully performed by Tuffier.[19]

Dr. George Fowler (New York) has devised a means of rectal implantation of the ureters in a valve-like manner, for exstrophy of the bladder.[20] The so-called malarial inflammation of the testis is believed by Le Dentu to be really elephantiasis of the organ characterised by distension of the lymph vessels.[21] Some twenty cases of acute torsion of the cord are described by S. H. Perry, who finds that the condition is twice as frequent on the right side as on the left.

An interesting case of spurious hermaphroditism was seen by Mr. Andrew Clark, a male-widow, married sixteen years, with a testicle in the left inguinal region. Menstruation had been regular from a vagina, but there was no uterus.[22]

**Venereal Diseases.**—St. Clair Thomson strongly advises mercurial inunction for tertiary syphilis of the nose and throat. Surgeon-Major Lambkin has practised and recommends intra-muscular injections in the treatment of syphilis owing to its certainty and its convenience.[23] Dr. Ahman has found gonococci in the blood in gonorrhœa, and Rendu is of opinion that to cure gonorrhœal rheumatism it is necessary to cure the discharge. The treatment of gonorrhœa by the silver salt "Protargol" has the strong support of Neisser.[24] In connection with the treatment of gonorrhœa by urethral and intravesical irrigations, reference may be made to an article by Dr. F. C. Valentine, in which such an apparatus is illustrated.[25]

[7] Med. Rec., Feb. 5, 1898. [8] Annals of Surgery, Jan. 7, 1898. [9] Annals of Surgery, April, 1898. [10] Brit. Med. Jour., April 2, 1898. [11] Brit. Med. Jour., Feb. 19, 1898. [12] Med. Rec., Mar. 26, 1898. [13] Med. News, Feb. 12, 1898. [14] Med. Rec., Mar. 26, 1898. [15] Med. Rec., Mar. 5, 1898. [16] Lancet, Jan. 22, 1898. [17] Annals of Surgery, Mar., 1898. [18] Practitioner, Feb., 1898. [19] Brit. Med. Jour. Epit., April 30, 1898. [20] Amer. Jour. Med. Sci., Mar., 1898. [21] Revue de Chirurgie, Jan., 1898. [22] Brit. Med Jour., Mar., 12, 1898. [23] Brit. Med. Jour., Feb. 19, 1898. [24] Clinical Jour., April 6, 1898. [25] New York Med. Rec., April 16, 1898.

